# Effect of HTC and Water-Leaching of Low-Grade Biomasses on the Release Behavior of Inorganic Constituents in a Calcium Looping Gasification Process at 650 °C

**DOI:** 10.1021/acs.energyfuels.4c02833

**Published:** 2024-08-22

**Authors:** Markus Kopsch, Florian Lebendig, Elena Yazhenskikh, Álvaro Amado-Fierro, Teresa Centeno, Michael Müller

**Affiliations:** † Institute of Energy Materials and Devices (IMD-1), Forschungszentrum Jülich GmbH, Wilhelm-Johnen Straße, Jülich 52428, Germany; ‡ Instituto de Ciencia y Tecnología del Carbono (INCAR), CSIC, Francisco Pintado Fe 26, Oviedo 33011, Spain

## Abstract

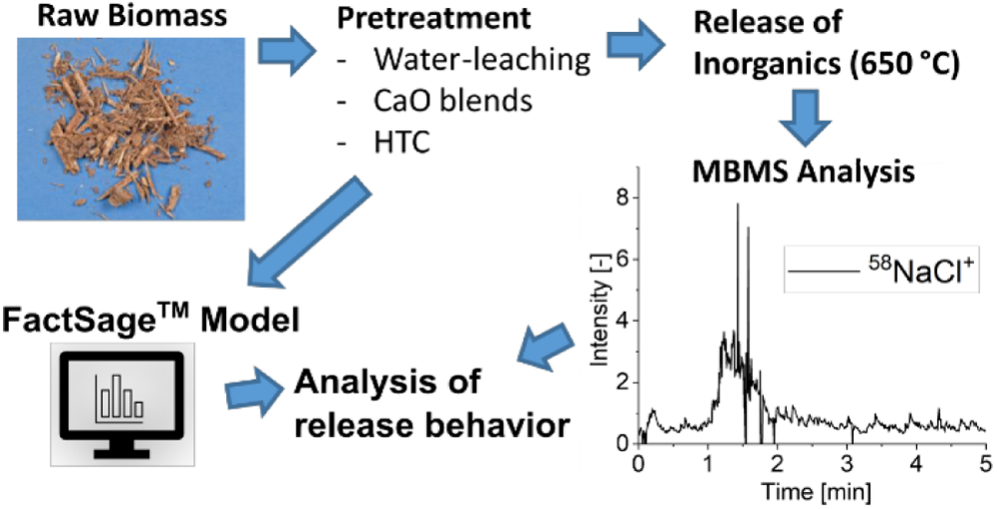

The release of alkali metals (K, Na) and nonmetals (S, Cl) during a calcium looping (CaL) gasification process of waste derived-hydrochars, water-leached samples, and CaO-biomass blends was investigated. Special attention was paid to biomasses that are not particularly promising for gasification requirements but have a large occurrence in Europe, including Grape Bagasse, Organic Fraction of Municipal Solid Waste (OFMSW), Green Waste, and Out-of-use woods from construction debris and discarded furniture. The release experiments were performed at 650 °C in a flow channel reactor to investigate the behavior of inorganic trace substances. Hot-gas analysis was performed by Molecular Beam Mass Spectrometry (MBMS). Thermodynamic equilibrium calculations via FactSage indicate H_2_S, carbonyl sulfide (COS), KCl, NaCl, and HCl as the main inorganic impurities. Thus, the focus of the experiments was placed on these species. It was found that the concentrations of trace elements released during gasification at 650 °C, such as H_2_S, SO_2_, KCl, and NaCl, are hardly affected by intense water-leaching. In contrast, carbonaceous materials from hydrothermal carbonization exhibit a higher concentration of trace potassium substances (K, KCl, and K_2_Cl^+^). When biomass samples are combined with CaO, the total amount of inorganic trace compounds (K, Na, and S compounds) in the resulting syngas could be decreased.

## 1 Introduction

According to the German government’s coalition agreement of 2021, energy use is expected to reach 680–750 terawatt hours (TWh) by 2030.^1^ Of this, 80% is to come from renewable energy sources. Germany exceeded its 2020 renewable energy target of 35% by supplying 46% of its electricity from renewable sources. In 2000, this figure was just 6%. Biomass energy is an important supplement to wind and solar power. Because bioenergy is easily stored, it can be used whenever it is needed, especially in the absence of wind and sunlight. Renewable energies contributed 16% to primary energy consumption in 2021. Biomass energy continues to be the largest contributor to renewable energy, with a share of 52%, followed by wind power (just under 28%), solar energy (photovoltaics and solar thermal, 12%), hydropower (4%), and geothermal energy (4%).

**Figure 1:**
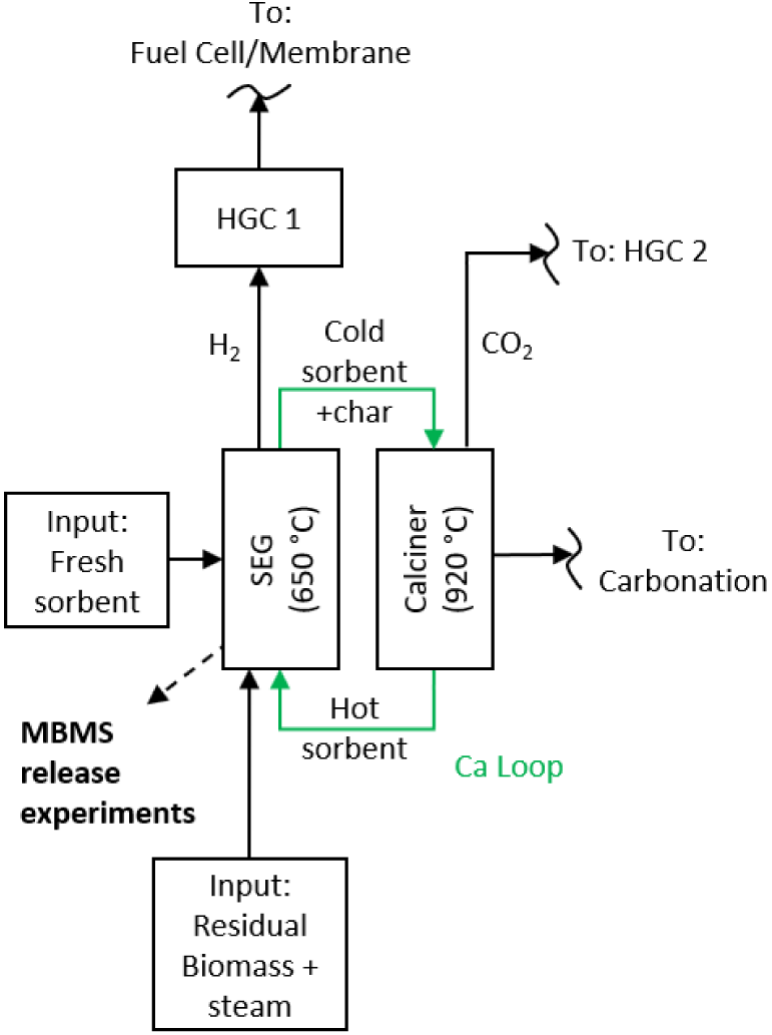
CO_2_ capture via Ca-looping gasification (SEG = sorption enhanced gasification; HGC = hot gas cleaning).

One project that seeks to develop an advanced approach to convert energy from biomass into biofuel and on-demand power production by integrating biomass gasification technology is the European GICO-Project.^2^ In the GICO-Process, Ca-looping gasification (Figure 1) is applied to produce a hydrogen-rich syngas, which will be used in a fuel cell after hot gas cleaning (HGC). The sorption enhanced gasifier (SEG) is operated at 650 °C. As these temperatures are below the conventional operating temperatures of most fluidized bed gasifiers, the release behavior of inorganic trace substance has hardly been investigated, making further experimental investigations necessary.

The use of CaO as a primary sorption material in the gasification reactor (SEG) reduces the amount of CO_2_ by forming CaCO_3_ (see Reaction 1^1^), which is then fed, together with the produced char, into the calcination unit. Reducing CO_2_ with CaO influences the water gas shift (WGS) equilibrium (see Reaction 2^2^) in the sense of higher CO conversion and H_2_ output according to Le Chatelier’s principle. This has been demonstrated in some thermodynamic modeling studies.^3,4^
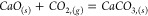
1
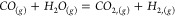
2

CaO has the ability to not only reduce CO_2_ but also has the potential to absorb gaseous sulfur and chlorine species. Through forming CaS, CaSO_4_, and CaCl_2_, various trace substances such as H_2_S, SO_2_, carbonyl sulfide (COS), and HCl can be significantly decreased.^5,6^ CaS can be further oxidized to CaSO_4_ in the calciner. Additionally, low concentrations of H_2_S also result in lower levels of COS, as shown in Reactions 3^3^–7.
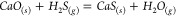
3
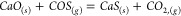
4
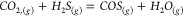
5
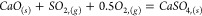
6
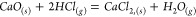
7

Trace elements such as sulfur (S), chlorine (Cl), potassium (K), and sodium (Na) can potentially have a significant impact on both the gasifier and downstream components.^7^ They can lead to high temperature corrosion (e.g., chlorination and sulfidation), catalyst deactivation, and deposition by agglomeration.

Biomass upgrading is a promising approach to counteract ash-related issues and to facilitate or enhance the utilization of low-grade biomass fuels in thermochemical bioenergy applications. Several biomass upgrading approaches, e.g., torrefaction,^8−10^ microwave pretreatment,^11−13^ and leaching^14−16^ are considered as potential process steps for thermochemical conversion of biomass utilization.

Pretreating the biomass through water-leaching and drying can significantly reduce emissions for straw gasification, particularly for water-soluble alkalis.^17−20^ Leaching by rain can reduce purification efforts for grassy biomasses. However, leaching has a minimal effect on woody biomasses, since alkalis are primarily organically bound.^21^ Extraction experiments with empty fruit bunch (EFB) have shown that both chlorine and potassium concentrations can be significantly reduced by up to 80–90% after four consecutive extractions with water.^18,22^ Additionally, research indicates that water-leaching can increase the ash melting point by several hundred degrees Celsius.^16^ However, insoluble inorganic components cannot be removed through water-leaching as they are bound to active sites of lignin, cellulose, or hemicellulose. In order to remove these components, acidic environments like HCl are necessary.^23^

Cl can promote the release of potassium in the form of gaseous KCl.^24^ Potassium is thus released more as a result of the Cl content in fuels than potassium content. As confirmed by Molecular Beam Mass Spectrometry (MBMS), water-leaching successfully reduced the amount of alkali chlorides released from the fuel during conversion due to their significant reduction in the fuel.^18^ Consequently, their condensation downstream the gasifier shifted from temperatures above their melting points to temperatures below; thus, contamination should be less significant. Using batch-type experiments, it was found that the potassium release behavior depends on the Si/K fuel molar ratio. Higher Si content results in less K being released, which is beneficial for preventing fouling. When Si/K ratios are very high (e.g., sewage sludge), potassium is embedded well in the slag, resulting in extremely low K concentrations in the gas.^18,25^

Hydrothermal carbonization (HTC) is a novel technology that has gained increasing interest in recent years as a pretreatment strategy for biodegradable (wet) wastes.^26−30^ In this process, the waste is treated in the presence of water at moderate temperatures (160–300 °C) and a self-generated pressure. Under these conditions, water acts as a reagent, solvent, and catalyst, resulting in rapid degradation of the biopolymers and solubilization of some of the inorganic components. Furthermore, this sustainable treatment ensures waste sterility and has the potential to degrade emerging contaminants and endocrine disruptors.

The HTC process involves biomass feedstocks undergoing a variety of reactions, such as hydrolysis, dehydration, decarboxylation, polymerization, and aromatization.^31,32^ The solid product generated by HTC, the so-called hydrochar, is enriched with carbon and, depending on the feedstock composition and HTC operation conditions, has properties similar to peat, lignite, or high-volatile bituminous coals.^33,34^ Increased aromaticity and hydrophobicity improves the drying of hydrochar^33^ and generates energy densification, leading to increased heating value. In addition, hydrochar exhibits better fuel qualities due to its decreased nitrogen and chlorine content.^35^ The behavior of chlorine, nitrogen, and phosphorus during biowaste hydrothermal carbonization has been well studied.^36,37^

Sorption enhanced gasification (SEG) is usually operated at gasification temperatures between 600 and 800 °C. The present laboratory gasification experiments were carried out at 650 °C. The hot gases were analyzed for trace compounds using Molecular Beam Mass Spectrometry (MBMS). Particular attention was paid to alkali metals (K, Na) and nonmetals (S, Cl), as these play an important role in the release behavior of a wide range of biomasses. Furthermore, the concentration of trace species in the gas was predicted by thermodynamic equilibrium calculations using FactSage. The knowledge gained in this work about the type and amount of inorganic substances set free during gasification can help to select suitable biomasses and a suitable pretreatment method for low-temperature gasification.

## 2 Modeling and Experimental Section

### 2.1 Thermodynamic Modeling of the GICO Process with FactSage

Since knowledge of inorganic trace substance concentrations in syngases from (pretreated) biomasses is fundamental for the removal of trace substances, a model for describing the release of trace substances in syngases was created. Due to the large variety of syngas components, biomass gasification is a complex chemical process. In FactSage, a thermodynamic calculation tool, thermodynamic equilibria in various chemical systems can be calculated depending on chemical composition, temperature, and pressure by minimizing the Gibbs energy of the system. FactSage is a product of the companies Thermfact (Canada) and GTT-Technologies (Germany).^38^ The in-house developed oxide database GTKT^39^ and the commercial database SGPS were used for the calculations. In the case of duplicate species, GTKT was given higher priority.

**Figure 2:**
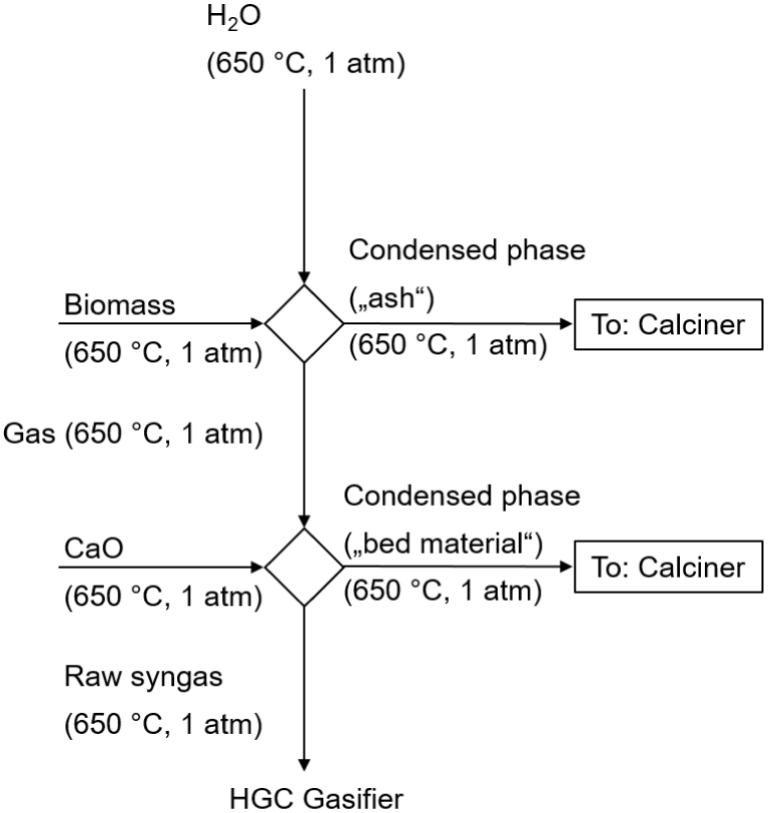
Schematic representation of the gas release calculations via FactSage.

As shown in Figure 2, the GICO model consists of two equilibrium reactors and phase separators represented by squares. The equilibrium reactors are connected to each other and to the environment via material flows, represented by arrows. The model begins with the introduction of the two input streams, water and biomass, into the gasifier. Water is added until all of the elemental C from the biomass has been oxidized to carbon monoxide and carbon dioxide at 650 °C under atmospheric pressure. With the help of a phase separator, the gas phase is then separated from the solid phase so that only the gas phase is considered in the CaO sorption calculation. In this way, the considerably faster reaction between the gas and CaO should take precedence over the solid–solid reactions between the ash components of the biomass and the sorbent. The amount of CaO used in the modeling was determined using the in-built transition function in FactSage. This function can be used to determine the amount of CaO above which no further change in the gas phase occurs. This approach simulates a sufficient residence time and amount of CaO for the complete conversion of all potential reactants in the fluidized bed reactor.

After the gas-CaO reaction, the gas and condensed phases are separated once again. The following work gives a summary of the achievable gas stream purities before and after the CaO reaction. The composition of the biomasses used in the model is shown in Table 1. The values from the elemental and ICP-OES analyses (Tables 4–7 in the experimental section) were normalized to 100% so that the concentration ratio of the investigated elements in the biomasses and in the model is identical.

**Table 1:** Biomass Compositions in the Simulation [wt %]^a^

biomass	C	H	N	S	Cl	O	Al	Ca	Fe	K	Mg	Na	P	Si
green waste	39.1	5.33	1.54	0.20	0.23	36.22	1.07	2.29	0.94	1.14	0.28	0.10	0.00	11.58
green waste hydrochar	47.6	5.28	0.99	0.11	0.11	35.08	0.63	1.93	0.65	0.51	0.15	0.06	0.00	6.89
OFMSW	42.0	5.76	2.82	0.24	0.82	36.85	0.36	4.67	0.36	1.01	0.55	0.77	0.73	3.05
OFMSW hydrochar	48.6	6.09	2.51	0.18	0.49	31.76	0.27	5.30	0.23	0.76	0.26	0.48	1.05	1.98
out-of-use woods	46.7	6.19	1.96	0.09	0.06	44.41	0.04	0.20	0.05	0.07	0.03	0.06	0.00	0.13
out-of-use woods hydrochar (whey)	48.0	5.97	1.42	0.10	0.32	42.09	0.04	0.56	0.19	0.38	0.07	0.28	0.00	0.55
out-of-use woods hydrochar	51.5	5.77	2.40	0.10	0.11	37.86	0.08	0.51	0.16	0.08	0.10	0.29	0.00	1.03
grape bagasse	48.8	5.94	2.38	0.18	0.003	37.66	0.03	0.34	0.17	3.94	0.12	0.01	0.31	0.09
grape bagasse hydrochar	57.6	6.09	2.32	0.18	0.01	30.62	0.07	0.38	0.12	2.41	0.07	0.02	0.00	0.12

a The values are based on the 100% normalization of the values found in Tables 4–7.

### 2.2 Pretreatment and Characterization of Biomass Samples

Four different types of biomass wastes were used for the release experiments. Emphasis was placed on feedstocks that are not highly favored for gasification due to their high inorganic content. However, biomasses with a large occurrence in Europe including grape bagasse, organic fraction of municipal solid waste (OFMSW), green waste, and out-of-use woods from construction debris and end-of-life-furniture have been selected. The release behavior of both the untreated biomass and the three pretreated biomasses was investigated:

1) Solids from HTC process
2) Water-leached samples
3) Samples mixed with CaO

The hydrochars were obtained by subjecting the diverse feedstocks at 195 °C and 13.2 bar for 3 h in a HTC pilot reactor of 1 m^3^-capacity. The biomass/water ratio was 1:4 and the water already present in biomass was considered as part of the reaction medium.

To achieve greater profitability and a lower environmental impact based on the reduction of water consumption and the elimination of two wastes simultaneously, a cohydrothermal carbonization of out-of-use woods was approached by using whey instead of water as a reaction medium. The samples were dried to mass constancy at 105 °C and vacuum-sealed for shipment.

To increase the specific surface and thus improve the mass transfer between the fuel and water during leaching, each sample was milled and fractionated to a diameter of 0.2 mm. All biomass samples (untreated and hydrochars) were washed twice. Each washing cycle lasted 1 h. In each case, 500 mL of deionized water were added to 50 g fuel sample in PET bottles. The bottles were then placed on a roller so that the contents were mixed. After each cycle, the sample was vacuum filtered by using a water aspirator. After the washing cycle, the biomass was dried to a constant mass at 105 °C.

50 mg per experiment was used when dealing with the pretreated (pure) biomass samples. For the mixture release experiments, however, CaO was mixed 1:1 (by mass) with each biomass sample. In contrast to the pure biomass experiments, 100 mg of sample material were used in order to keep the amount of biomass constant. By keeping the amount of CaO constant (50 mg), the C (S, Cl, etc.) to CaO ratio changes for different pretreatment methods. Therefore, all untreated and pretreated samples (water-leached, HTC, HTC + water-leached) were mixed with CaO to compare them with their counterparts without CaO. In this way, the influence of CaO on the release can be investigated. The molar ratio of C in the biomass to CaO is between 0.35 (grape bagasse hydrochar + water-leached) and 0.67 (green waste). Accordingly, CaO is available substoichiometrically for the reaction of CO_2_ to CaCO_3_ (see Table 2).

**Table 2:** Molar Ratio of Biomass C and CaO [mol_C_/ mol_CaO_]

biomass	*n*_C (Biomass)_/ *n*_CaO_
green waste	0.68
green waste (water-leached)	0.60
green waste hydrochar	0.48
green waste hydrochar (water-leached)	0.45
OFMSW	0.55
OFMSW (water-leached)	0.52
OFMSW hydrochar	0.47
OFMSW hydrochar (water-leached)	0.45
out-of-use woods	0.47
out-of-use woods (water-leached)	0.45
out-of-use woods hydrochar	0.43
out-of-use woods hydrochar (water-leached)	0.41
out-of-use woods hydrochar (treated with whey)	0.46
out-of-use woods hydrochar (treated with whey + water-leached)	0.43
grape bagasse	0.44
grape bagasse (water-leached)	0.41
grape bagasse hydrochar	0.38
grape bagasse hydrochar (water-leached)	0.35

In contrast to the experiments, CaO was added stoichiometrically in the modeling so that the gas concentrations no longer changed. This method was used to estimate which species are important with regard to typical problems such as high-temperature corrosion, slagging, or fouling. With the experimental substoichiometrical approach, the risk that the residence time of the biomass samples is too short for complete gasification can be represented.

Each sample was chemically characterized. An elemental analysis was performed for C, H, N, S, and O. For the major ash forming elements (e.g., K, Ca, Na, etc.) optical emission spectroscopy combined with inductively coupled plasma (ICP-OES) was used. The filtrate for both washing cycles was collected and used for the quantification of Ca, K, Mg, Na, P, Si, and Cl using ICP-OES. The analysis was carried out by the Central Institute of Engineering, Electronics and Analytics (ZEA-3) at the Forschungszentrum Jülich. In order to be able to better compare the influence of HTC and washing on the biomass components, the results of the different biomasses were presented separately. The results are presented in Tables 4–7,7 of the Results and Discussion section.

### 2.3 Release Experiments Using Molecular Beam Mass Spectrometry (MBMS)

Inorganic gaseous species released during gasification were measured using Molecular Beam Mass Spectrometry (MBMS). The experimental setup allowed the analysis of hot gases from various biomass-derived feedstocks under gasification-like atmospheres. This technique employed common mass spectrometry to analyze the mass-to-charge ratios (*m*/*z*) in an electromagnetic field.

**Figure 3:**
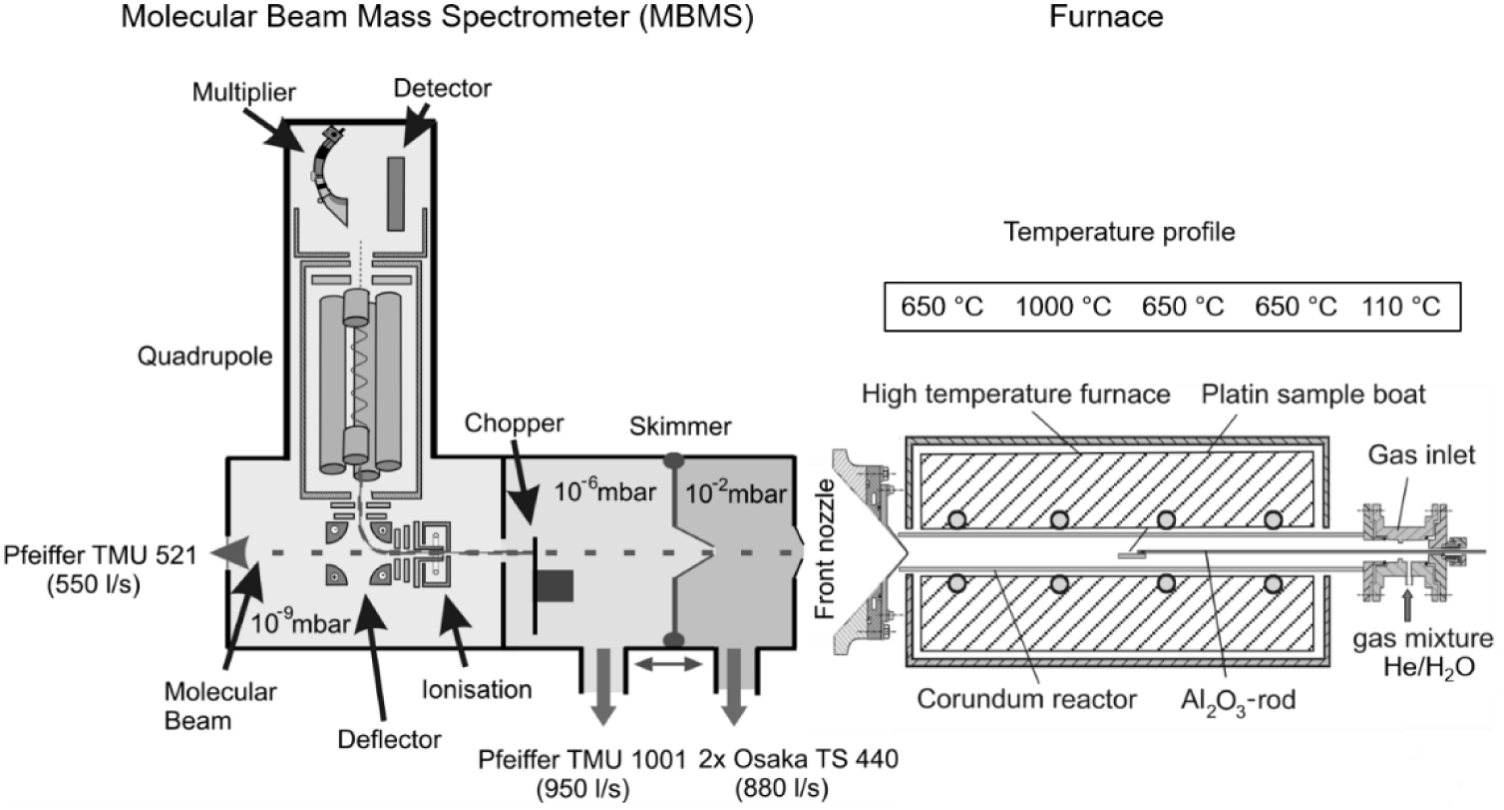
Experimental setup for release experiments.

Experiments were conducted under gasification-like conditions at 650 °C, using a four-zone furnace with an alumina tube connected to the MBMS nozzle as the gas inlet. The sample was gasified in the first two zones at 650 °C, while the third zone was set to 1000 °C to prevent condensation and to crack all formed hydrocarbons, enabling the study of inorganic species only. A visualization of the setup is given with Figure 3. An explanation of the functioning of the MBMS used is omitted here, as this has already been provided in numerous places.^40,41^

**Figure 4:**
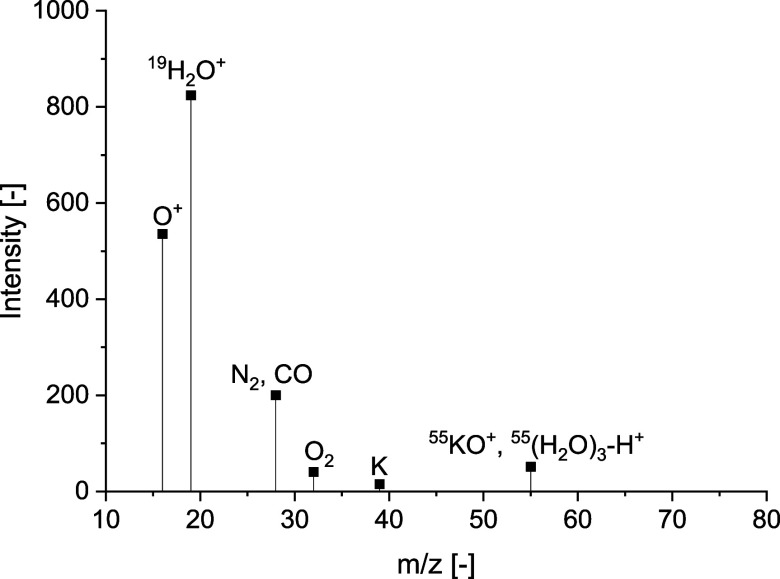
Intensities of mass spectra recorded in a vacant tube (reference measurement).

A continuous spectrum of masses 1 to 200 was recorded during an empty tube measurement. Gas components with an expected high concentration (and correspondingly high intensity) were excluded from the measurement in order to increase the sensitivities of the trace substances. Figure 4 shows the spectrum after removing masses 18 (H_2_O), 37 ((H_2_O)_2_–H), and 44 (CO_2_).

Intensity-time profiles of ^19^H_2_O^+^/^19^OH^+^, ^34^H_2_S^+^, ^35^Cl^+^, ^36^HCl^+^, ^38^HCl^+^, ^39^K^+^, ^55^KO^+^, ^58^NaCl^+^, ^60^COS^+^, ^64^SO_2_^+^, ^74^KCl^+^, and ^113^K_2_Cl^+^ were recorded and normalized to the ^19^H_2_O^+^ base level signal for quantification. Each sample was measured five times, and the averages were used for error calculations and semiquantitative analysis. A gas consisting of 20 vol % H_2_O and 80 vol % He was used throughout the measurement campaign. The total gas flow was set to 4 l/min for each experiment. 50 mg of fuel were gasified in Al_2_O_3_-sample boats in a single run and kept in the furnace for 5 min. The results obtained can be compared semiquantitatively using bar graphs.

## 3 Results and Discussion

### 3.1 Modeling Results of Biomass Gasification (SEG)

#### 3.1.1 Gasification without CaO

This section summarizes all calculations carried out with FactSage. As described in the previous section, the calculations include the steam gasification calculations and the syngas-CaO calculations. In order to better understand the influence of primary sorption by means of CaO in the gasification unit, the calculated concentrations before and after the CaO reaction are listed in this section. Since CaO does not only have an influence on the CO_2_ concentration in the syngas but also reacts with other gas components (e.g., H_2_S, HCl, etc.) the trace substance behavior must also be considered.

**Figure 5:**
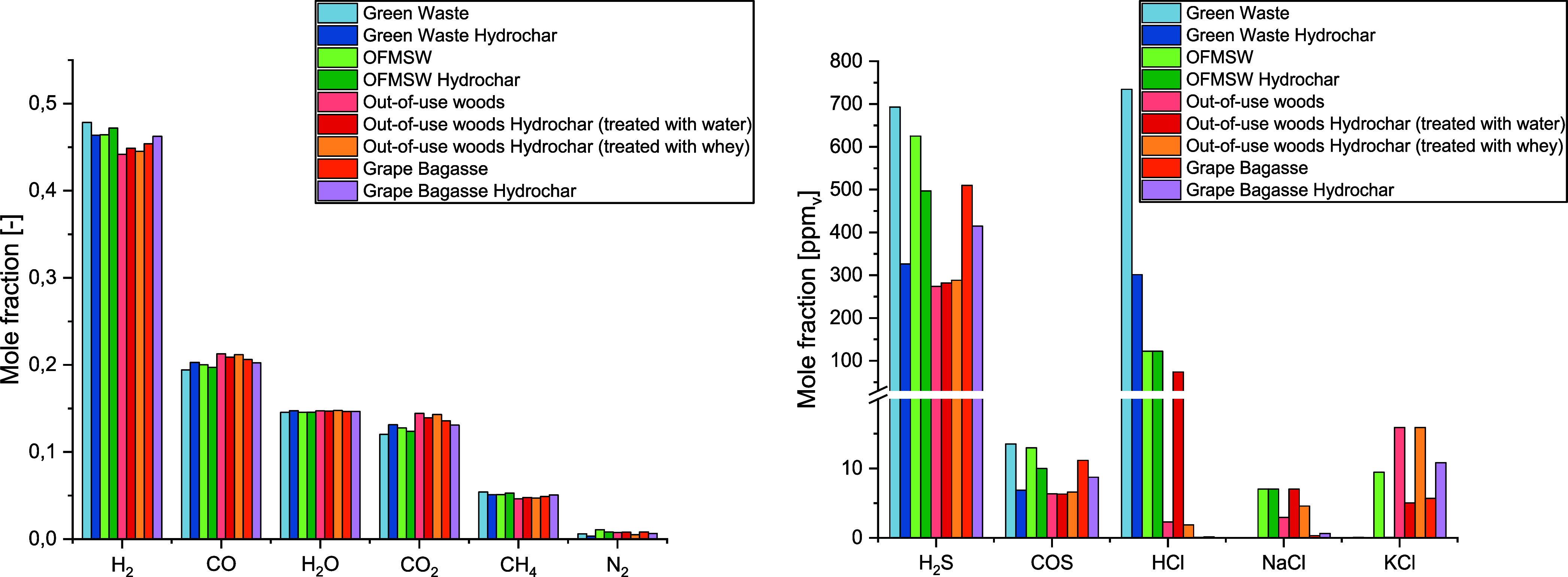
Concentrations of the main components (l.) and concentrations of alkali, chlorine, and sulfur species (r.) in the simulated syngas during gasification without CaO (650 °C, 1 atm).

**Figure 6:**
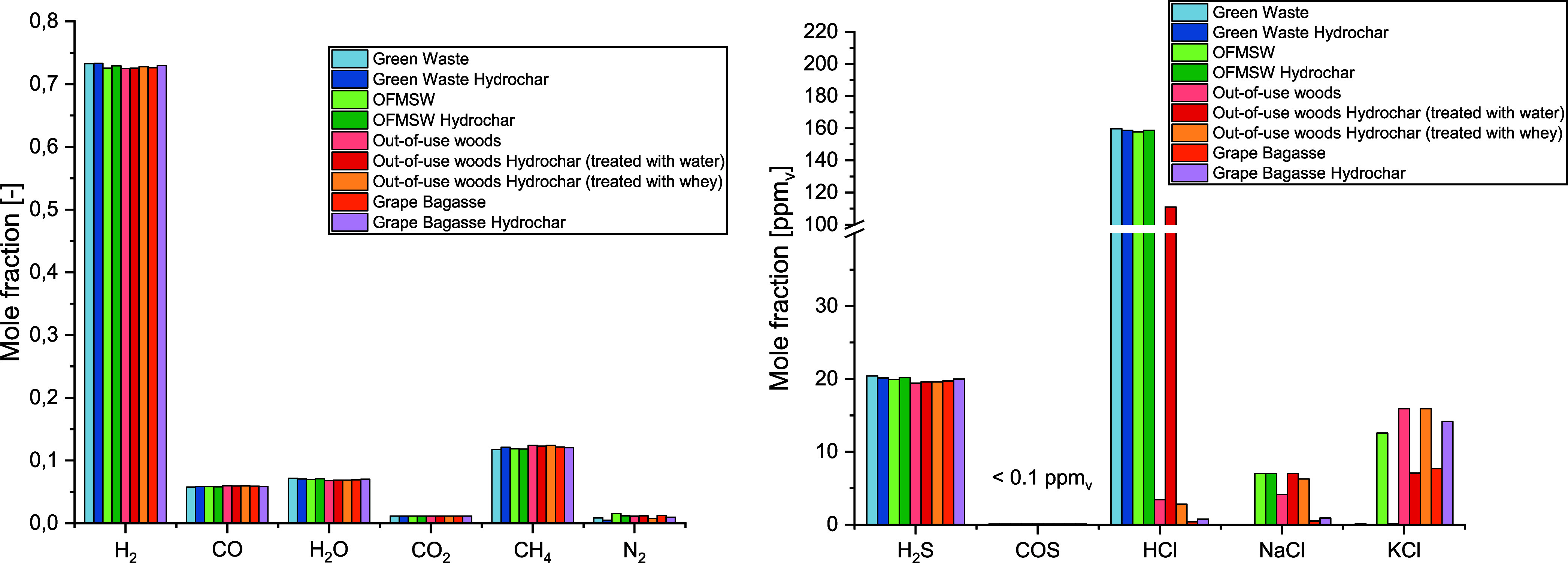
Syngas compositions of the main components (l.) and concentrations of alkali, chlorine, and sulfur species (r.) in the simulated syngas during gasification after CaO reaction (650 °C, 1 atm).

By reference to the input conditions of the gasifier described before and the biomass composition presented in Table 1, the simulation results on the syngas compositions are shown in Figures 5 and 6. Due to the high differences in concentration, the main components (H_2_, CO, H_2_O, CO_2_, CH_4_, and N_2_) of the syngas are listed separately from the trace substances. Regarding the main components produced during the steam gasification at 650 °C, Figure 5 (l.) shows a fairly homogeneous composition of the syngas, irrespective of the biomass. On average, the syngas before the reaction with CaO consists of approximately 45.9% H_2_, 20.0% CO, 14.7% H_2_O, 13.3% CO_2_, 5.0% CH_4_, and 0.7% N_2_.

The presentation of trace substances in Figure 5 (r) is limited to those alkali, sulfur, and chlorine species that have a concentration above 0.10 ppm_v_. Due to the immense differences in concentration of the individual trace species, the dependence on the biomass is directly recognizable: Those syngases with high concentrations of a trace species usually exceed those with low concentrations many times over. Thus, the HCl concentration in the syngas from green waste (734 ppm_v_) and from its hydrochar (301 ppm_v_) clearly exceeds that of grape bagasse or grape bagasse hydrochar with less than 1 ppm_v_ according to the Cl content in the biomass (see Table 1). The high Si content in some biomasses favors the forming of solid compounds with K, Na, and Ca (e.g., CaSiO_3_, KAlSi_3_O_8_, NaAlSi_3_O_8_), leaving Cl for the reaction to form HCl. Thus, the low HCl concentrations of out-of-use woods, out-of-use woods hydrochar (treated with whey), grape bagasse, and grape bagasse hydrochar result partly from the low Si concentrations of the biomasses.

Due to the reducing atmosphere in the gasifier, oxidation of the sulfur bound in the biomass is almost completely prevented. As a result, the majority of sulfur appears in the form of hydrogen sulfide (H_2_S), as can be clearly seen in Figure 5 (r.). The H_2_S concentration is ranging from 281 ppm_v_ in the syngas from out-of-use woods hydrochar (treated with water) to over 692 ppm_v_ in the syngas from green waste. Due to the equilibrium reaction with CO_2_ (see Reaction 4^4^), a small amount of sulfur is also bound in carbonyl sulfide (COS). Accordingly, COS concentrations range from 6.3 ppm_v_ in syngas from out-of-use woods hydrochar (treated with water) to 13.5 ppm_v_ in syngas from green waste.

In general, the potassium load in biomasses is higher than the sodium load. This often leads to KCl concentrations higher than those of NaCl in the syngas.

#### 3.1.2 Influence of CaO on the Syngas Composition

Since CaO is used as the primary sorption material for CO_2_ in the GICO fluidized bed gasification (SEG), the concentrations not only of the main components but also of the sour gas components are changing according to Reactions 1^1^ and 3–7.

Figure 6 (l.) shows that, similar to the syngas concentrations before the CaO reaction, the concentrations of the main components in the gas phase of the different biomasses hardly differ. The CaO reacts with CO_2_ in the gas phase to form CaCO_3_ and thus, lowers the CO_2_ concentration in the syngas to approximately 1%. This is the lowest CO_2_ concentration that can be achieved with CaO since CaO is added in excess in the simulation, meaning that the equilibrium in Reaction 1^1^ cannot be shifted further to the right side. The low CO_2_ concentrations shift the equilibrium according to Reaction 2^2^ to higher H_2_ concentrations (approximately 73%). The elemental nitrogen in the biomass forms mainly inert N_2_ (1%). Moreover, the syngases consist of approximately 12% CH_4_, 6% CO, and 7% H_2_O.

As described earlier, CaO also has an influence on the HCl concentration. Since the CaO was added in excess in the simulation, the concentrations shown in Figure 6 are the minimum achievable concentrations. Concentrations of approximately 20 ppm_v_ are reached for H_2_S. The HCl concentrations can be reduced to approximately 160 ppm_v_ with CaO. Since the HCl concentrations of all out-of-use woods and grape bagasse samples were already low after the steam gasification, they cannot be further reduced. The concentrations for NaCl and KCl were found to increase for those biomasses, where the syngas was not saturated, due to the reduction of the gas volume after the CaO reaction.

### 3.2 Experimental Results

#### 3.2.1 Fuel Composition

The release behavior of inorganics was investigated under gasification-like conditions. To comprehend the impact of various pretreatment methods, a semiquantitative analysis was carried out. Both, the pretreated (water-leached biomass, HTC samples, CaO-biomass mixtures) and the untreated biomasses were investigated under gasification-like conditions using a MBMS. Thus, the influence of the pretreatment and the influence of CaO on the release behavior can be determined.

**Figure 7:**
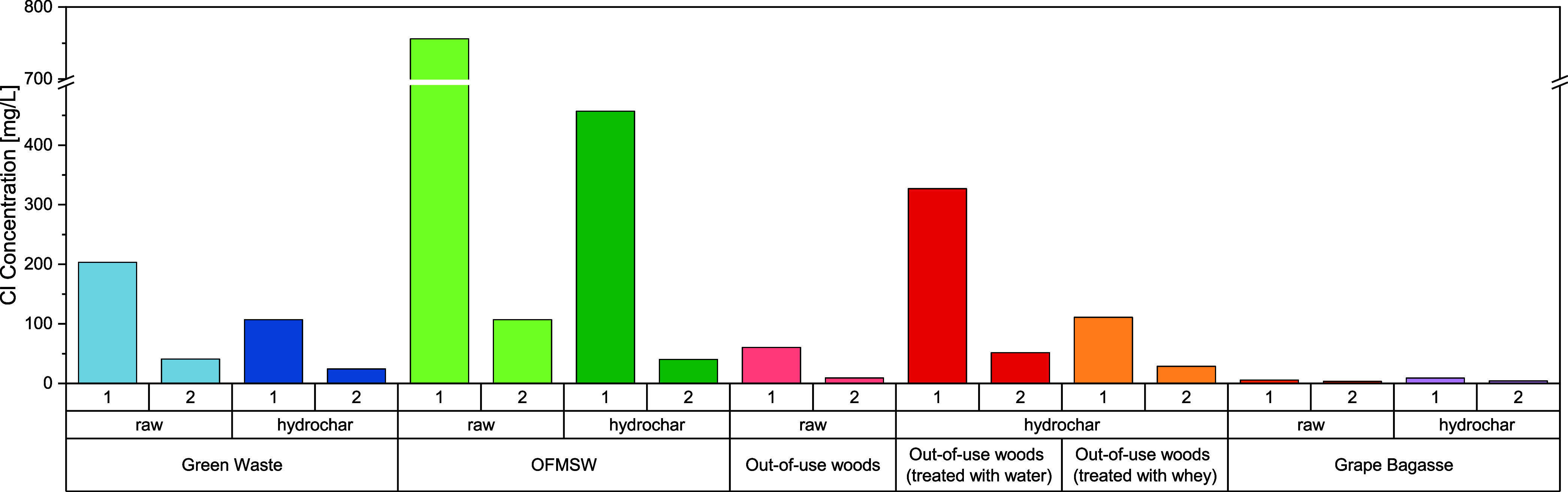
Comparison of Cl detected in the washing water after the first and second washing cycles.

As described previously, the amount of impurities observed during gasification can be reduced by pretreatment of the fuel. Two washing cycles were performed on the samples. Figure 7 shows the Cl concentration of the filtrate collected during the wash cycles.

Chlorine was detected in the washing water for all biomass samples. The concentration after the first washing cycle was correspondingly higher than that after the second washing cycle. Furthermore, Cl was also clearly washed out of the hydrochars (HTC samples), which had previously been exposed to water during preparation.

The results of the S, Cl, K, and Na analysis of the untreated feedstocks and the results for the water-leached feedstocks are listed in Tables 4–7. The effect of water-leaching on the Cl content of the fuels is noticeable. However, no effect is observed on the Si content due to its insolubility. Since potassium is mainly present in the fuel as highly soluble salts such as KCl, water-leaching also reduces the K content.

The biomass contents of S, Cl, K, and Na affect the syngas composition. High contents might result in higher concentrations of H_2_S, respectively, SO_2_, KCl, NaCl, and HCl.

Overall, the analyzed hydrochars (HTC samples) demonstrate a lower content in alkali, alkaline earth, and phosphorus compounds compared with the corresponding raw fuels, likely due to leaching during hydrothermal carbonization (HTC). Additionally, the hydrochar also exhibits a 2–22% higher carbon concentration compared to the raw fuels, highlighting the carbon enrichment that occurs during the hydrothermal carbonization process. On the other hand, the sulfur concentration in the hydrochar has generally remained relatively constant compared to the raw fuels. Table 3 shows the percentage of sulfur in the raw fuel that is retained in the hydrochar.

**Table 3:** Retained Sulfur in Hydrochar [wt %]

biomass	retained sulfur in hydrochar
green waste	42.8
OFMSW	52.0
out-of-use woods	93.1
out-of-use woods hydrochar (treated with whey)	98.8
grape bagasse	72.4

The ash resulting from the MBMS experiments was collected and analyzed using the X-ray diffraction method. BaSO_4_ and TiO_2_ were detected in the out-of-use wood samples. Synthetic BaSO_4_ is used as a component of white pigments for paints. Titanium dioxide pigments are also used in products such as paints and coatings. However, BaSO_4_ is poorly soluble in water. BaSO_4_ particles could therefore only have physically separated from the biomass during the washing process.

#### 3.2.2 Release Behavior of Untreated Biomasses Under Gasification-Like Conditions

**Figure 8:**
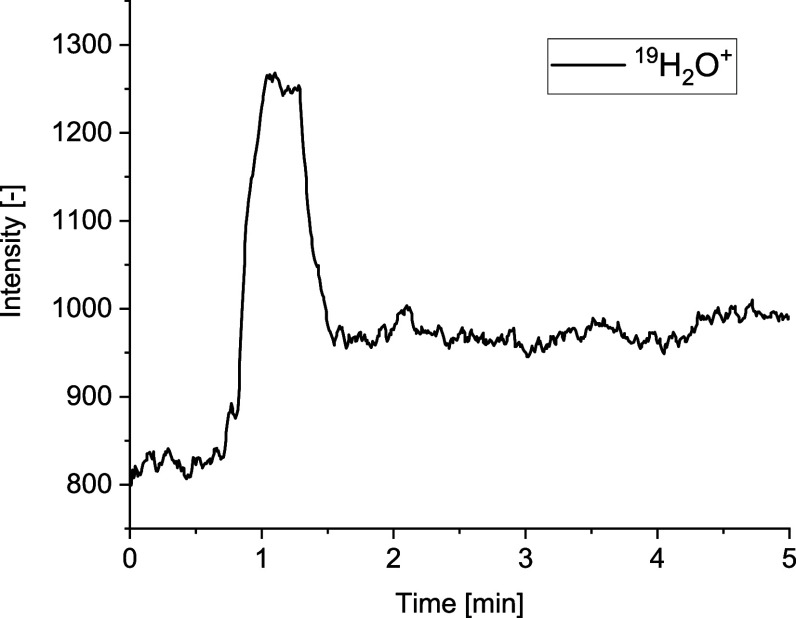
Intensity-time profile of ^19^H_2_O^+^ (*m*/*z* = 19) of grape bagasse at 650 °C in 20 vol % H_2_O and 80 vol % He (V̇_tot_ = 4 l/min).

Since the multiplier occupancy of the MBMS and thus the intensity sensitivity decrease after several experimental runs, the intensities of the species investigated in this work must be related to a value that includes the sensitivity losses. Figure 8 shows the intensity-time profile of the ^19^H_2_O^+^ signal, which served as the base level for normalizing the different signal areas. A short peak identifying the volatile trace compounds can be seen immediately at the beginning of each measurement. Devolatilization reactions are typically characterized by high kinetic rates.^20^ The signal after this peak is at a higher level than that of the basic signal before the measurement. This might indicate a kinetic inhibition of the complete H_2_O release due to the high H_2_O content (20%) in the gas.

On the contrary, char gasification and ash reactions are characterized by low kinetic rates. Unlike in other investigations,^42,43^ a second, broader peak cannot be detected or can only be detected to some extent. This is mainly due to the low temperature of 650 °C used in the experiments, where many inorganically bound elements that are set free during char gasification have relatively low vapor pressures, especially.

**Figure 9:**
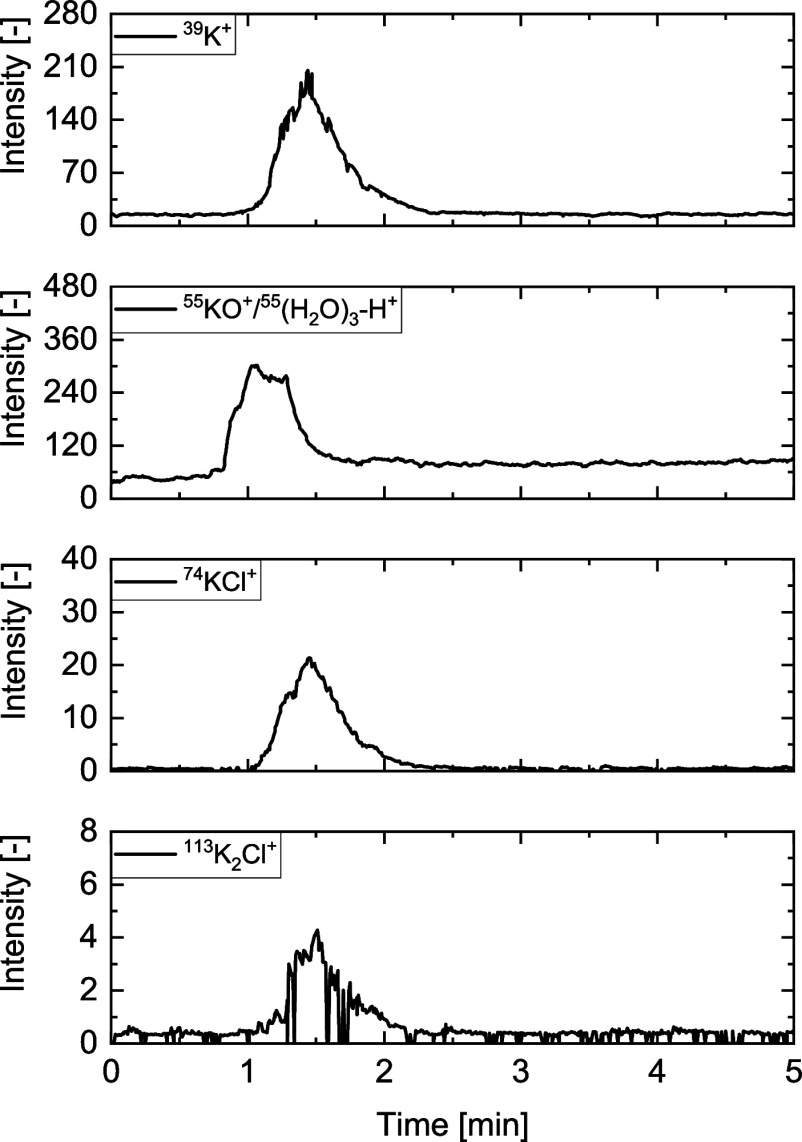
Intensity-time profiles of ^39^K^+^ (*m*/*z* = 39), ^55^KO^+^/^55^(H_2_O)_3_-H^+^ (*m*/*z* = 55), ^74^KCl^+^ (*m*/*z* = 74), and of ^113^KCl^+^ (*m*/*z* = 113) of grape bagasse at 650 °C in 20 vol % H_2_O and 80 vol % He (V̇_tot_ = 4 l/min).

In recent publications,^18,40^*m*/*z* = 39 was assigned to ^39^K^+^. To rule out the possibility that fragment *m*/*z* = 39 belongs to ^39^NaO^+^ or ^39^C_3_H_3_^+^, the intensity-time profiles of potassium-containing species, i.e., ^39^K^+^ (*m*/*z* = 39), ^55^KO^+^ (*m*/*z* = 55), ^74^KCl^+^ (*m*/*z* = 74), and ^113^K_2_Cl^+^ (*m*/*z* = 113) from grape bagasse, are plotted in Figure 9. It is evident that the signals of ^39^K^+^, ^74^KCl^+^, and ^113^K_2_Cl^+^ exhibit similar release duration, start, and peak shape. Nonetheless, there may be some small overlap with other species.

An exception is *m*/*z* = 55. Preliminary experiments showed that the measurement of ^56^KOH^+^ (*m*/*z* = 56, often fragmented to KO^+^ with *m*/*z* = 55) can only be done with the inclusion of larger errors due to superposition by a water cluster (^55^(H_2_O)_3_H^+^) and its isotopes. The release of the component found at *m*/*z* = 55 occurs earlier than that of the other potassium-containing components and simultaneously with water at signal 19. Furthermore, no clear signal could be detected at *m*/*z* = 56. Therefore, KOH cannot be detected independently.

The intensity-time profile of ^58^NaCl^+^ of an untreated grape bagasse sample is later compared with an intensity time profile of the grape bagasse-CaO blend (see Figure 15). Although the NaCl concentration was only slightly above the detection limit of the ICP-OES (0.01 wt %), a signal can still be distinguished from the background. Nevertheless, due to the low concentration, there is an increased collapse of the signals.

The dip of the signal can partly lead to difficulties in the evaluation as the integration limits are not clearly identifiable. Grape bagasse has a ^39^K^+^ intensity 50 times higher than the example presented for ^58^NaCl^+^.

**Figure 10:**
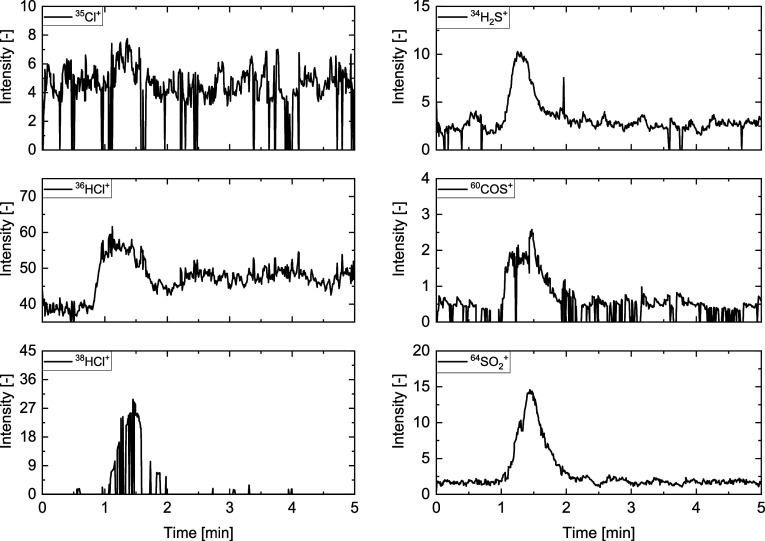
Intensity-time profiles of ^35^Cl^+^ (*m*/*z* = 35), ^36^HCl^+^ (*m*/*z* = 36), ^38^HCl^+^ (*m*/*z* = 38), ^34^H_2_S^+^ (*m*/*z* = 34), ^60^COS^+^ (*m*/*z* = 60), and ^64^SO_2_^+^ (*m*/*z* = 64) of grape bagasse at 650 °C in 20 vol % H_2_O and 80 vol % He (V̇_tot_ = 4 l/min).

Besides KCl and NaCl, another problematic compound in gasification is HCl. Figure 10 shows the intensity-time profiles for ^35^Cl^+^, ^36^HCl^+^, and ^38^HCl^+^, and detected sulfur-containing species are ^34^H_2_S^+^, ^60^COS^+^, and^64^SO_2_^+^. Although the experiments were carried out under gasification-like conditions, SO_2_ is the sulfur component with the highest concentration. In the gasification atmosphere, H_2_S is typically the dominating S-compound, as there is not enough oxygen for oxidation to SO_2_. The simultaneous presence of H_2_S and SO_2_ may indicate that small amounts of oxygen were present during the experiments, despite the high flow rate of helium. Furthermore, H_2_O could react with H_2_S during the experiment to form some SO_2_ (see Reactions 8^8^ and 9).
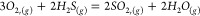
8
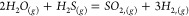
9

Since H_2_S is only present in the ppm_v_ range under such conditions, the amount of unwanted O_2_ is unavoidable and may also occur in such small quantities in industrial gasifiers.

The levels of the ^35^Cl^+^ and ^38^HCl^+^ signals are not adequate for evaluation in this instance. The main signal of HCl at *m*/*z* = 36 appears to be obscured by the water cluster at *m*/*z* = 37.

#### 3.2.3 Influence of HTC and Water-Leaching on the Release Behavior Under Gasification-Like Conditions

Consistency in the intensity-time profiles of the gas components was observed for both the hydrochar (HTC) and water-leached samples, as illustrated in the earlier section: Once more, only single peaks were detected, without any double peaks or plateaus, for the masses that were presented. Furthermore, the profiles of the different potassium components are matching in shape and thus verify the existence of potassium-containing components.

**Figure 11:**
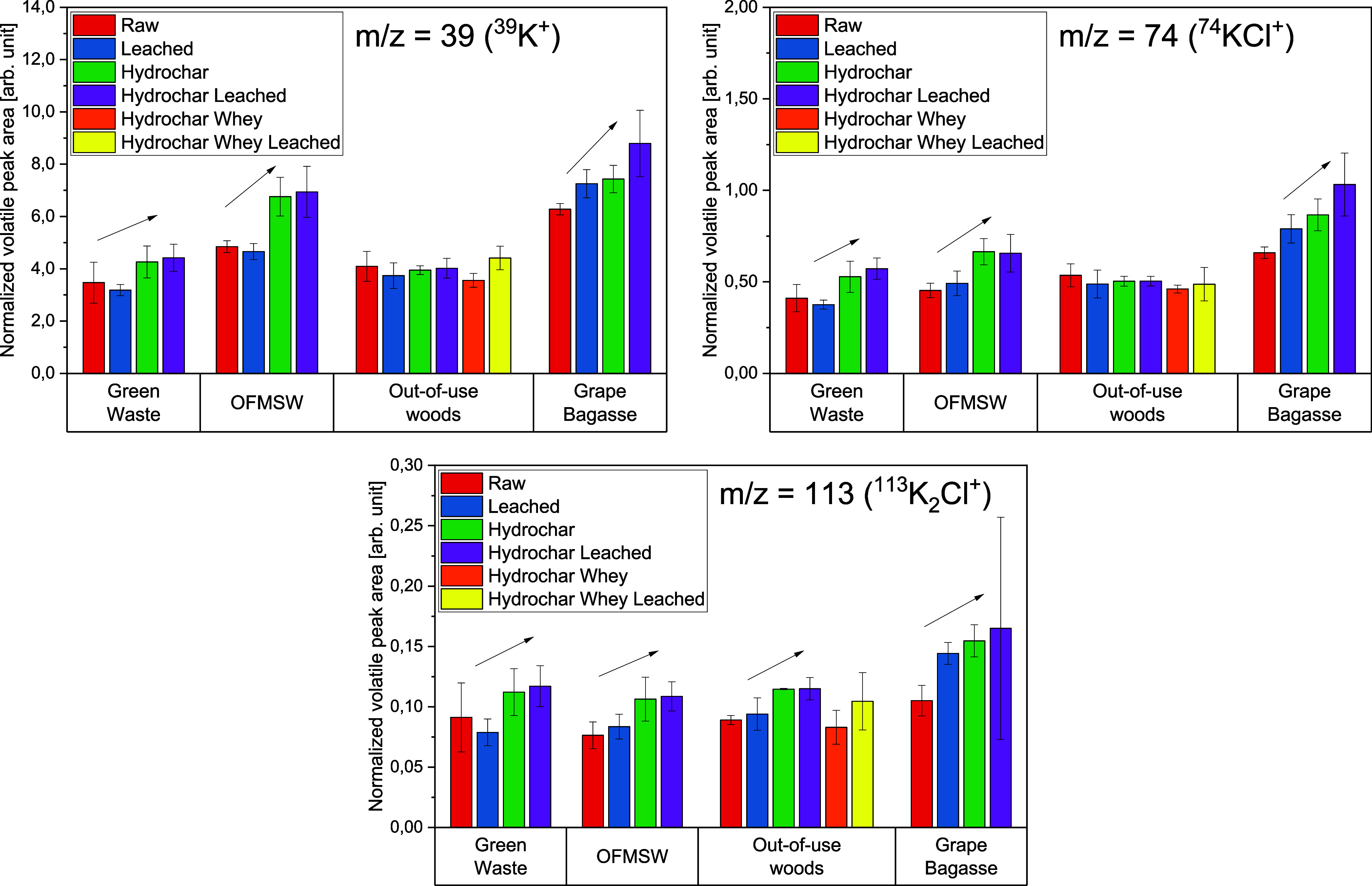
Averaged, normalized peak areas of potassium species released during the devolatilization phase (*n* = 5).

**Figure 12:**
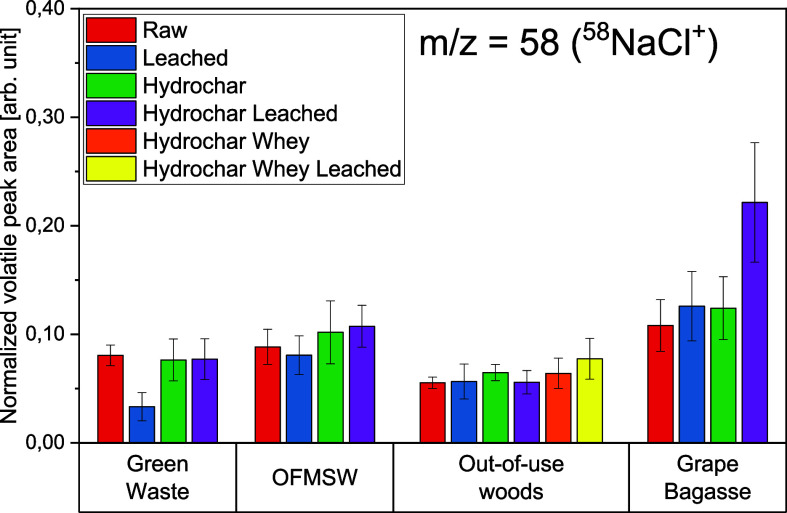
Averaged, normalized peak areas of NaCl released during the devolatilization phase (*n* = 5).

**Figure 13:**
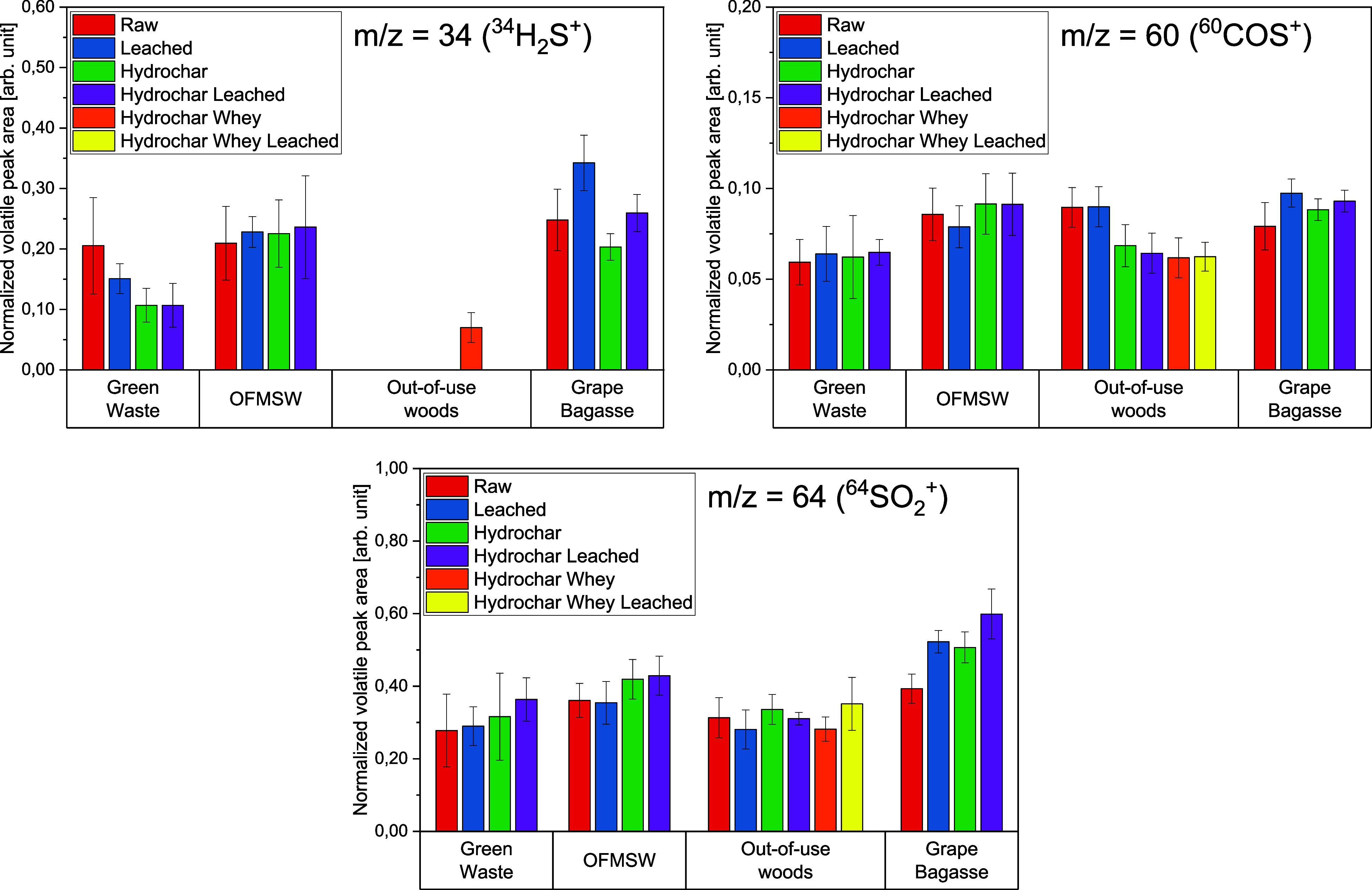
Averaged, normalized peak areas of sulfur species released during the devolatilization phase (*n* = 5).

Figures 11–12,13 show the volatile peak areas of different species normalized to the base signal of *m*/*z* = 19. Five measurements per sample were performed. Only masses that were significantly above the background noise are evaluated here.

In general, the results agree well with the chemical characterization results (see Tables 4–5,6,7, i.e., biomasses with a high potassium or sulfur content (e.g., grape bagasse) release more of the associated species (e.g., KCl, H_2_S, or SO_2_).

**Table 4:** Ultimate Analysis of Raw and Pretreated Green Waste Samples [wt %]

element	green waste	green waste (water-leached)	green waste hydrochar	green waste hydrochar (water-leached)
C	31.70	35.60	44.20	47.10
H	4.33	4.46	4.90	5.28
N	1.25	1.29	0.92	0.92
S	0.16	0.17	0.10	0.11
Cl	0.19	0.01	0.10	0.00
O	29.40	33.00	32.58	34.38
Al	0.87	0.91	0.59	0.70
Ca	1.86	1.93	1.80	1.49
Fe	0.76	0.71	0.60	0.55
K	0.92	0.57	0.48	0.22
Mg	0.23	0.25	0.14	0.09
Mn	0.02	0.02	0.01	0.00
Na	0.08	0.06	0.06	0.02
P	0.00	0.00	0.00	0.00
Si	9.40	5.49	6.40	2.70

**Table 5:** Ultimate Analysis of Raw and Pretreated OFMSW Samples [wt %]

element	OFMSW	OFMSW (water-leached)	OFMSW hydrochar	OFMSW hydrochar (water-leached)
C	38.70	40.80	45.60	47.30
H	5.31	5.76	5.71	5.72
N	2.60	2.82	2.35	1.98
S	0.22	0.23	0.17	0.18
Cl	0.76	0.02	0.46	0.01
O	33.96	32.67	29.78	27.49
Al	0.34	0.77	0.26	0.27
Ca	4.30	3.37	4.97	4.27
Fe	0.33	0.30	0.22	0.24
K	0.93	0.19	0.72	0.10
Mg	0.51	0.46	0.24	0.15
Mn	0.01	0.00	0.01	0.00
Na	0.71	0.16	0.45	0.12
P	0.68	0.58	0.98	1.31
Si	2.81	2.25	1.86	1.47

**Table 6:** Ultimate Analysis of Raw and Pretreated Out-of-Use Woods Samples [wt %]

element	out-of-use woods	out-of-use woods (water-leached)	out-of-use woods hydrochar (treated with water)	out-of-use woods hydrochar (treated with water, water-leached)	out-of-use woods hydrochar (treated with whey)	out-of-use woods hydrochar (treated with whey, water-leached)
C	45.50	47.80	49.30	52.70	46.50	49.80
H	6.03	6.19	5.52	5.80	5.78	6.07
N	1.91	1.94	2.30	2.04	1.37	1.24
S	0.09	0.00	0.10	0.00	0.10	0.00
Cl	0.05	0.01	0.11	0.01	0.31	0.01
O	43.25	42.71	36.24	36.31	40.75	40.04
Al	0.04	0.00	0.07	0.04	0.04	0.04
Ca	0.20	0.20	0.49	0.17	0.54	0.45
Fe	0.05	0.04	0.15	0.11	0.18	0.18
K	0.07	0.03	0.08	0.00	0.37	0.03
Mg	0.03	0.02	0.10	0.01	0.07	0.04
Mn	0.01	0.00	0.01	0.00	0.01	0.00
Na	0.06	0.04	0.27	0.00	0.27	0.03
P	0.00	0.00	0.00	0.00	0.00	0.00
Si	0.12	0.07	0.99	0.22	0.53	0.22

The potassium K^+^ peak areas of the biomasses seem to correlate with those of the KCl^+^ and K_2_Cl^+^ peaks. Higher KCl concentrations in the biomasses seem to lead to a proportional increase in the potassium signals. Compared to the untreated biomasses, most of the water-leached biomasses surprisingly show unchanged K^+^, KCl^+^, and K_2_Cl^+^ signal intensities despite demonstrably less potassium in the material studied. On the other hand, the hydrochar samples (HTC) show higher peak areas of potassium components even though the potassium concentrations in the hydrochar samples are lower compared to the raw samples. This is indicated by the arrows in Figure 11. The potassium components in the HTC samples (hydrochar) could be bound differently than in the noncarbonized biomasses resulting in a higher amount of volatile potassium. This can be attributed to various reactions that take place during HTC (e.g., polymerization, dehydration, hydrolysis, aromatization, etc.).^31,32^ However, a more in-depth analysis of the release behavior is outside the scope of this study.

The sodium concentrations in biomasses shown in Tables 4–7 are often lower compared to the concentrations of the potassium compounds. For many biomasses, sodium was either just at or slightly above the detection limit of the ICP-OES analysis or was not detectable at all. As a result, the peak areas detected by MBMS stood out only marginally from the background noise. Figure 12 shows the bar charts of the normalized peak areas of ^58^NaCl^+^. As can be seen therein, the intensity profiles are approximately one-tenth the size of those for ^39^K^+^.

Apart from the grape bagasse hydrochar (water-leached) sample, the profiles for the normalized volatile peak area are all close to each other. This indicates that the detection limit was reached. The different pretreatment methods are not reflected in an obvious trend here.

Unlike the potassium concentrations, the concentrations of the sulfur-containing compounds did not increase for the hydrochar samples (Figure 13). For the out-of-use woods samples, a H_2_S-signal could only be evaluated in one case due to the low sulfur concentrations in the samples.

The release of ^34^H_2_S^+^ was the highest for grape bagasse samples and the lowest for the out-of-use woods samples. These observations are in good accordance with the concentrations in the sample material (Tables 4–7). Grapes are considered to be extremely sensitive fruits. Conventionally, pesticides, including sulfur, are used to protect the soft skin from weather and fungi infestation.^44^ Fruit varieties such as grapes continue to draw attention due to traces of pesticides in samples.

**Table 7:** Ultimate Analysis of Raw and Pretreated Grape Bagasse Samples [wt %]

element	grape bagasse	grape bagasse (water-leached)	grape bagasse hydrochar	grape bagasse hydrochar (water-leached)
C	48.10	52.60	56.50	61.80
H	5.85	6.55	5.98	6.51
N	2.34	2.72	2.28	2.23
S	0.18	0.21	0.18	0.20
Cl	0.003	0.00	0.007	0.001
O	37.10	34.92	30.06	27.09
Al	0.03	0.02	0.07	0.02
Ca	0.33	0.34	0.37	0.44
Fe	0.17	0.12	0.11	0.06
K	3.88	1.22	2.37	0.30
Mg	0.12	0.02	0.07	0.00
Mn	0.01	0.00	0.02	0.00
Na	0.01	0.01	0.02	0.04
P	0.31	0.00	0.00	0.00
Si	0.09	0.08	0.12	0.14

#### 3.2.4 Influence of CaO on the Release Behavior Under Gasification-Like Conditions

**Figure 14:**
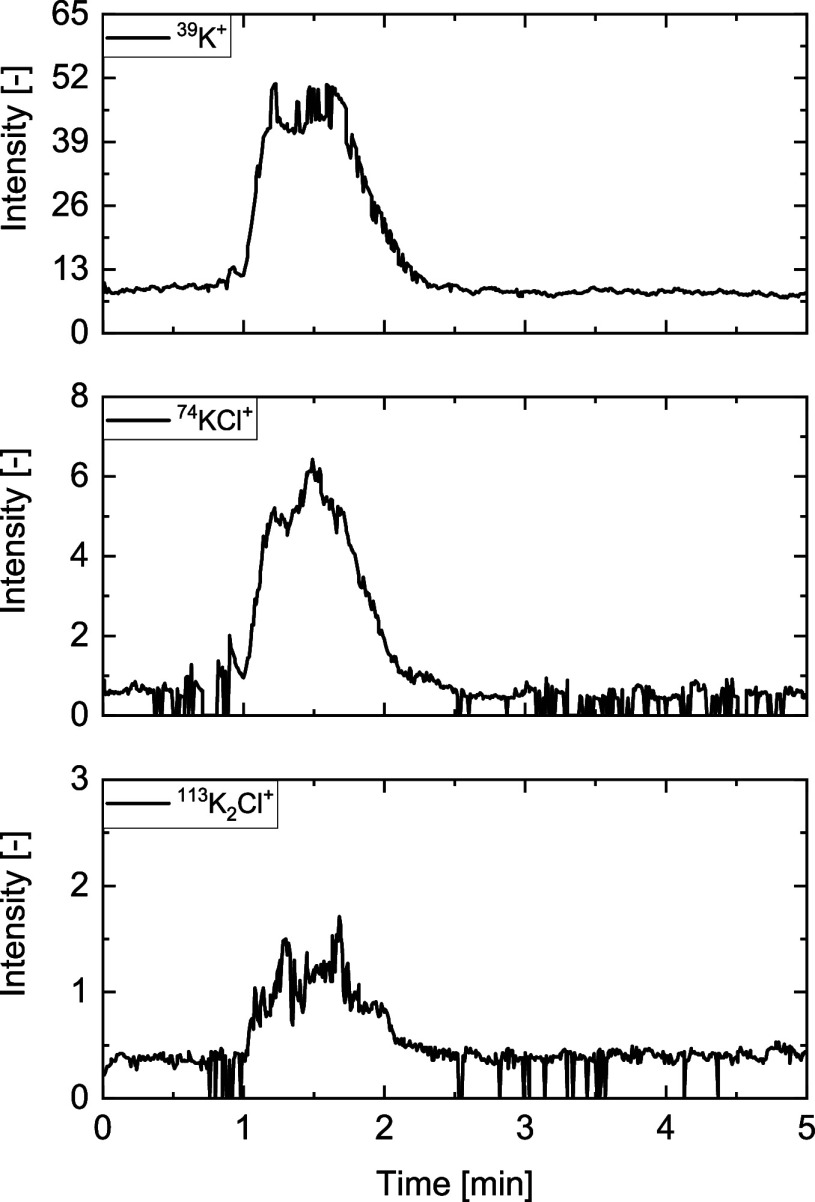
Intensity-time profiles of ^39^K^+^ (*m*/*z* = 39), ^74^KCl^+^ (*m*/*z* = 74) and^113^KCl^+^ (*m*/*z* = 113) of a CaO-grape bagasse mixture (50/50 wt %) at 650 °C in 20 vol % H_2_O and 80 vol % He (V̇_tot_ = 4 l/min).

To mimic the release behavior of the GICO gasifier, raw and hydrochar samples were blended with calcium oxide (CaO). The mass of the biomass sample was equal to the mass of the biomass samples from the previous section (50 mg). The CaO was added in the same amount (50/50 wt %). CaO was obtained by calcining CaCO_3_ at 920 °C for 5 h. CaCO_3_ could not be detected by XRD analysis after calcination anymore. Figure 14 shows the detected potassium components ^39^K^+^, ^74^KCl^+^, and ^113^K_2_Cl^+^ of the CaO-mixture samples. The ^39^K^+^ peak is not as sharp as the corresponding peak of the untreated biomass (see Figure 9). Other intensity-time profiles of the same sample indicate a second peak of similar height, which overlaps and forms a broader peak.

Potassium is released into the gas phase in two steps:^18^ First, potassium that is bound organically (in substances like lignin, cellulose, and hemicellulose) is released at temperatures of up to 500 °C. This process is not affected by the chlorine content in the fuel. In the second step, at temperatures above 500 °C, potassium is released at a full rate, mostly in the form of KCl and KOH, depending on the chlorine content of the fuel. Since CaO has a direct influence on the concentration of HCl (see Reaction 7^7^), the release behavior of potassium might also be affected.

Since the experimental parameters were maintained (flow rate, gas composition, and temperature), the difference in the intensity-time profiles is solely due to the CaO. XRD analyses were performed for all Grape Bagasse samples (untreated, water-leached, and blended with CaO) after the experiments. For the raw biomass, three potassium-containing phases could be identified (i.e., KHCO_3_, KH_4_(CO_3_)_3_**·**1.5H_2_O, and K_2_Ca(CO_3_)_2_), for the CaO mixture only one (i.e., K_2_Ca(CO_3_)_2_).

For the other two potassium-rich samples, green waste and OFMSW, a similarly altered peak shape can be detected. The addition of CaO seems to promote the release of the alkali components.

**Figure 15:**
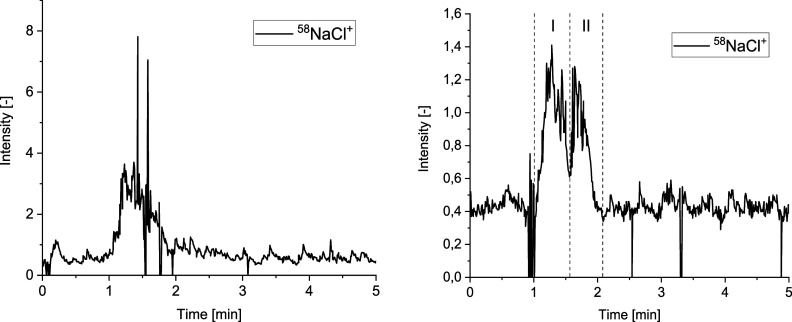
Intensity-time profiles of ^58^NaCl^+^ (*m*/*z* = 58) of grape bagasse (l.) and of ^58^NaCl^+^ of a CaO-grape bagasse mixture (50/50 wt %) at 650 °C in 20 vol % H_2_O and 80 vol % He (V̇_tot_ = 4 l/min).

Figure 15 shows the release behavior of the mass 58, which in previous work was mainly due to ^58^NaCl^+^.^45^ Similar to the release of various potassium components, the formation of a second peak can also be observed here.

**Figure 16:**
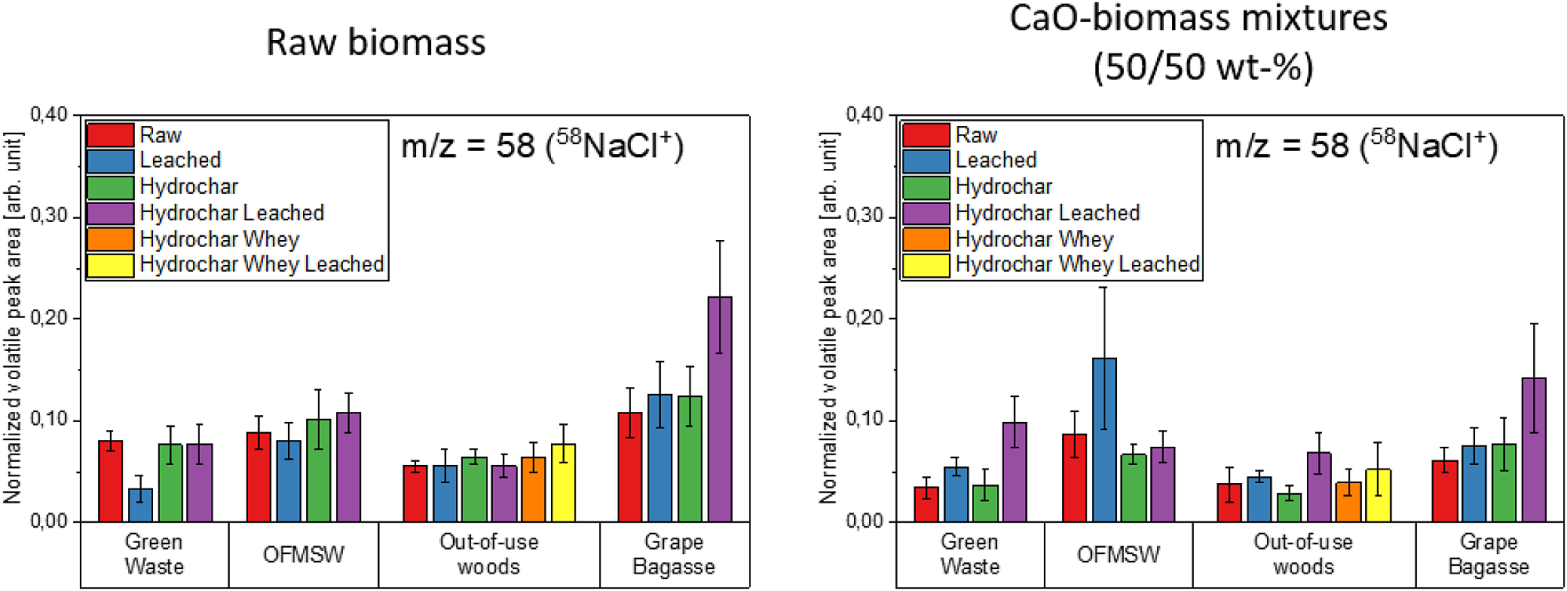
Averaged, normalized peak areas of NaCl released from biomass samples (l.) and from CaO-biomass mixtures (50/50 wt %) (r.) during devolatilization phase (*n* = 5).

**Figure 17:**
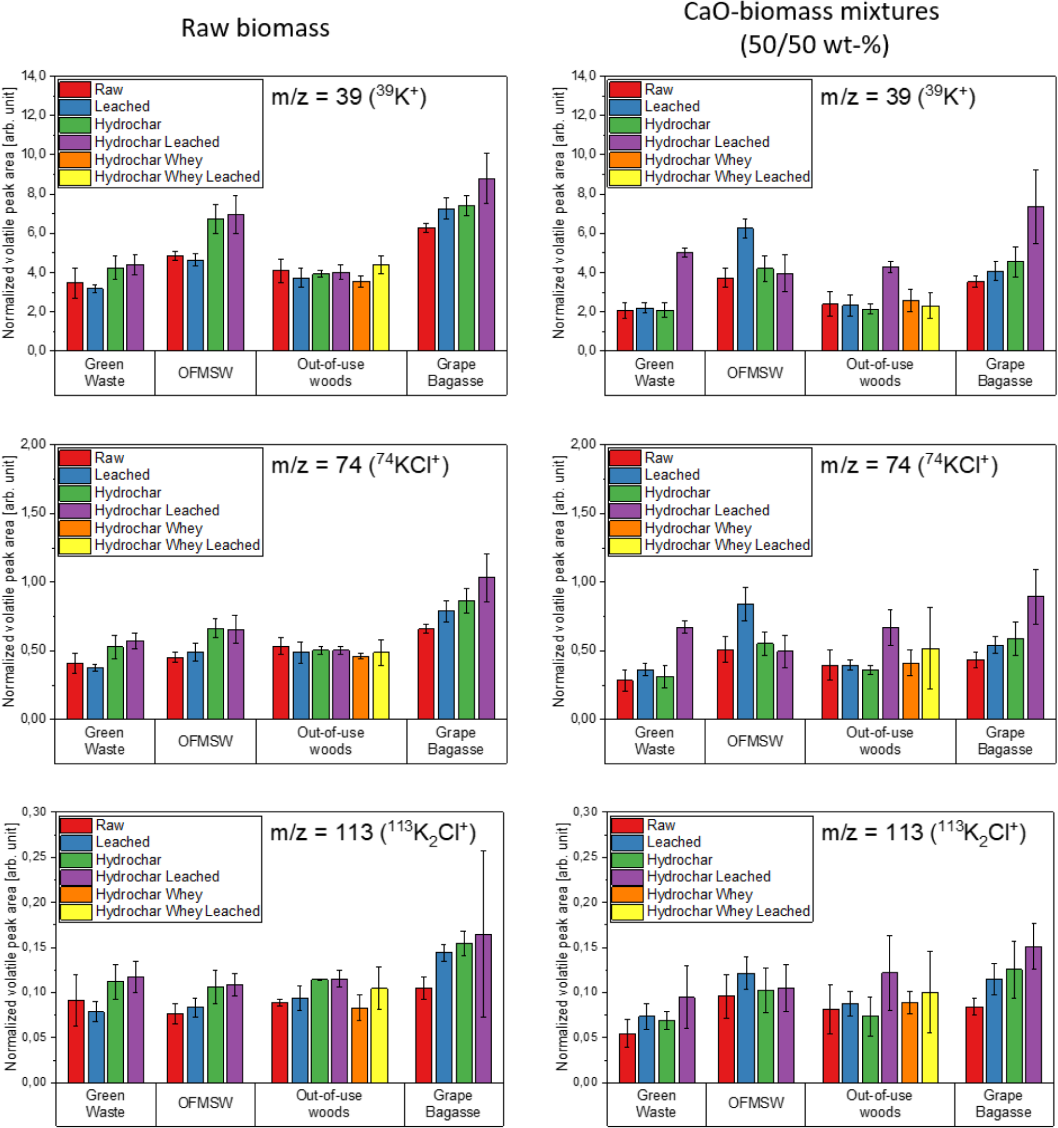
Averaged, normalized peak areas of potassium species released from biomass samples (l.) and CaO-biomass mixtures (50/50 wt %) (r.) during devolatilization phase (*n* = 5).

As already described, the amount of CaO remained constant during the experiments. Only the ratio of C (S, Cl, etc.) to CaO changed with the different pretreatment methods. For this reason, all untreated and pretreated samples were mixed with CaO in order to compare them with their counterparts without CaO. In this way, the sole influence of CaO on the release can be investigated. The molar ratio of C in the biomass to CaO is between 0.35 in the case of grape bagasse hydrochar + water-leached and 0.67 in the case of green waste (see Table 1). To allow a better comparison between the release behavior of the inorganic trace species of the untreated, water-leached, and HTC biomasses (l.) and the CaO-biomass mixtures (r.), the normalized peak areas of both experiments are placed side by side in Figures 16 and 17.

The normalized peak areas of the sodium and potassium components in the CaO containing experiments tend to be somewhat lower than in the release experiments without CaO. As already shown in Figure 10, the mass to charge ratios of 35, 36, and 38, which can be assigned to ^35^Cl^+^, ^36^HCl^+^, and^38^HCl^+^, are difficult to evaluate. However, the results show a tendency for the intensities mentioned to be lower in the CaO-biomass experiments than in the experiments without CaO. CaO may have reacted with HCl to give CaCl_2_.

**Figure 18:**
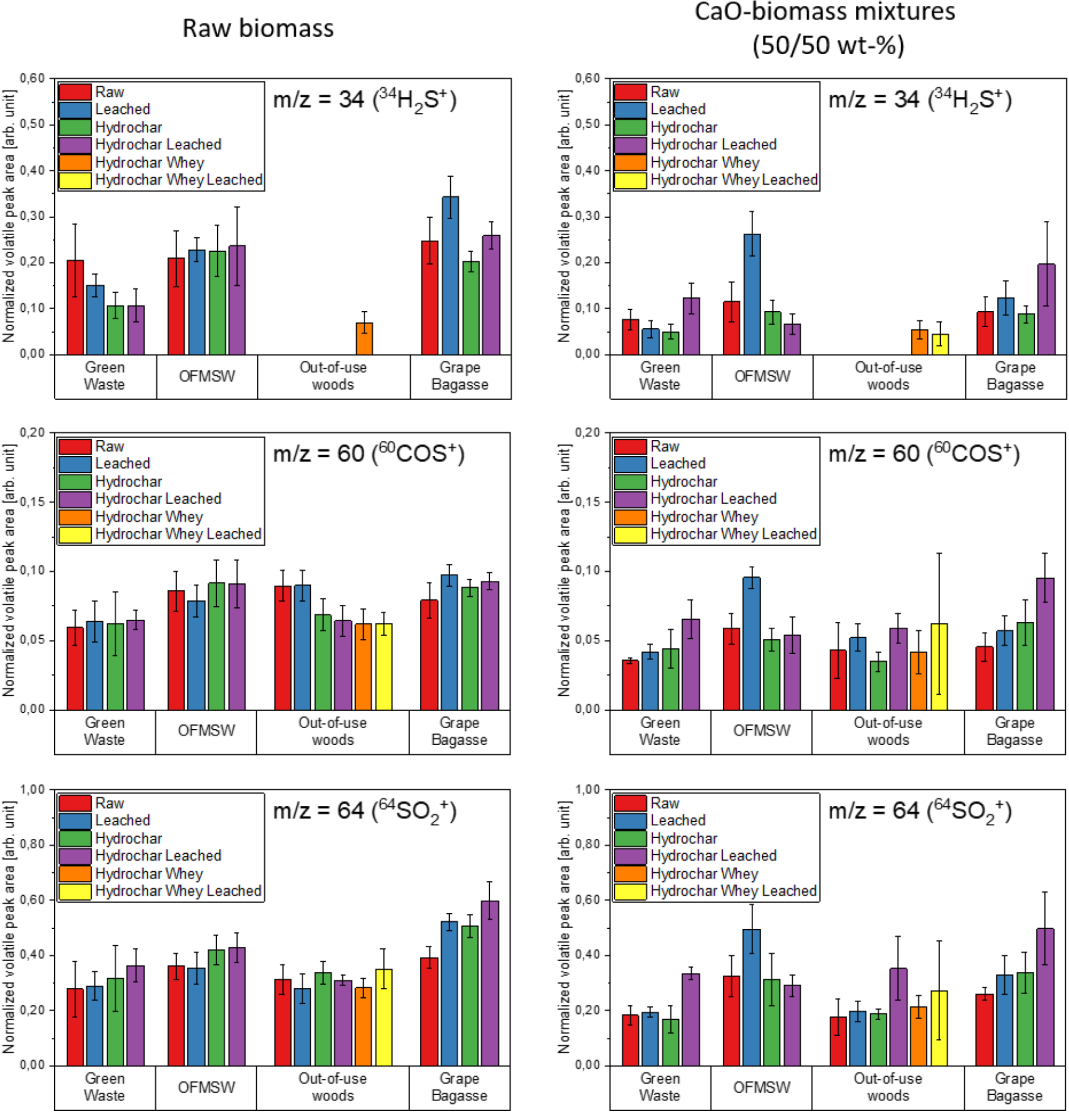
Averaged, normalized peak areas of sulfur species released from biomass samples (l.) and CaO-biomass mixtures (50/50 wt %) (r.) during devolatilization phase (*n* = 5).

In contrast to water-leaching and hydrochar (HTC samples), the CaO-biomass blends show a clear difference in the concentrations of the detected sulfur species (Figure 18). The rapid kinetics of the CaO and H_2_S reaction at 650 °C, which has been demonstrated in numerous studies,^46,47^ can be identified as the primary factor for the low intensities observed in this case. CaO can react directly with H_2_S, COS and SO_2_ (see Reactions 3^3^, 4 and 6) lowering their concentration. COS can also be decreased by the reduction of H_2_S (see Reaction 5^5^).

FactSage calculations at 650 °C for the sorption enhanced gasifier identified H_2_S, COS, HCl, and alkali chlorides KCl and NaCl as the main inorganic impurities. The release of the aforementioned inorganic trace substances was also clearly demonstrated in the release experiments. The modeling is therefore qualitatively suitable for predicting trace substances during release. In addition, when CaO was used, a significant decrease in H_2_S, COS, and HCl concentrations was seen in both the calculations and the experiments, while KCl and NaCl concentrations remained constant.

## 4 Conclusions

MBMS release experiments showed that intensive water-leaching hardly affects the concentrations of trace substances, such as H_2_S, SO_2_, KCl, or NaCl, released during gasification at 650 °C. It was anticipated that the peak areas of trace substances in the water-leached biomass samples would be smaller than those in the untreated samples. However, the concentrations of most of the normalized peak areas are within the standard deviation. This observation and conclusion could be due to the low gasification temperature of 650 °C used in this study.

The highest Cl concentration in the washing water was detected for OMFSW (>750 mg/L). However, OFMSW also has the highest Cl concentration in the biomass (0.76 wt %). The opposite is true for grape bagasse: there was hardly any Cl in the initial biomass examined (0.003 wt %), and the Cl concentration in the washing water was correspondingly low (<6 mg/L). It must be investigated whether the contamination should be removed from the washing water or as ash/coke and gas components in the gasifier.

On the other side, the concentration of potassium trace substances (K^+^, KCl^+^, and K_2_Cl^+^) released from the hydrochar samples is slightly higher (maximum 20% in the case of OFMSW) than that of the untreated and water-leached biomass samples, although ICP-OES measurements show that there are significantly lower inorganic trace substance concentrations in the hydrochar samples. This statement can be made for all biomasses except for out-of-use woods. Here, the bar graphs are not so clear. The release of potassium components, and thus the gas concentrations, may be increased for hydrochars at 650 °C, but for the nonhydrochar samples the potassium is more likely to remain in the solid phase. Whether it is advantageous to keep K components in the solid residue (ash, coke) in the gasifier or transfer them into the gas stream depends on the final design of the process (temperature profile of the reactor, type of hot gas cleaning, etc.).

Biomass samples that have been mixed with CaO show a second char/ash reaction peak (for potassium and sodium species) during gasification. These indicate that further alkali components were released. However, as the peak areas tend to be smaller, the use of CaO is a good way to reduce the concentration of inorganics in the syngas.
